# Hair Graying as an Evolutionary Checkpoint against Malignancy: a Stem Cell Perspective

**DOI:** 10.1007/s12015-026-11056-1

**Published:** 2026-01-10

**Authors:** Büşra Şensoy Gün

**Affiliations:** Central Research Laboratory Application and Research Center (BARUM), Bilecik Şeyh Edebali University, Bilecik, 11000 Turkey

**Keywords:** Hair graying, Canities, Melanocyte stem cells, Genotoxic stress, Oxidative stress

The human hair follicle serves as an exceptional, accessible model for studying tissue regeneration, neuroendocrine interactions, and stem cell aging. While hair graying (canities) is ubiquitously recognized as a cardinal sign of chronological aging, its biological significance extends far beyond cosmetic decline. Emerging research suggests that the loss of pigmentation is not merely a passive exhaustion of the pigmentary unit but represents an active, tightly regulated “checkpoint” mechanism. This perspective posits that the organism deliberately sacrifices pigmentation to preserve the genomic integrity of the stem cell pool, prioritizing survival and tumor suppression over cosmetic maintenance.

The pigmentation of the hair shaft is orchestrated by a dedicated population of Melanocyte Stem Cells (McSCs) residing in the bulge region, a specialized niche within the follicle. Unlike the continuous pigmentation seen in the epidermis, follicular melanogenesis is cyclical, tightly coupled with the hair growth phases (anagen, catagen, telogen). McSCs must coordinate self-renewal with differentiation into mature melanocytes that colonize the hair bulb. The irreversible depletion of this McSC reservoir is the fundamental etiology of graying. Current understanding identifies a complex “triad of destruction” driving this depletion: oxidative burden, neuroendocrine dysregulation, and genotoxic stress (Fig. 1).

**Fig. 1:**
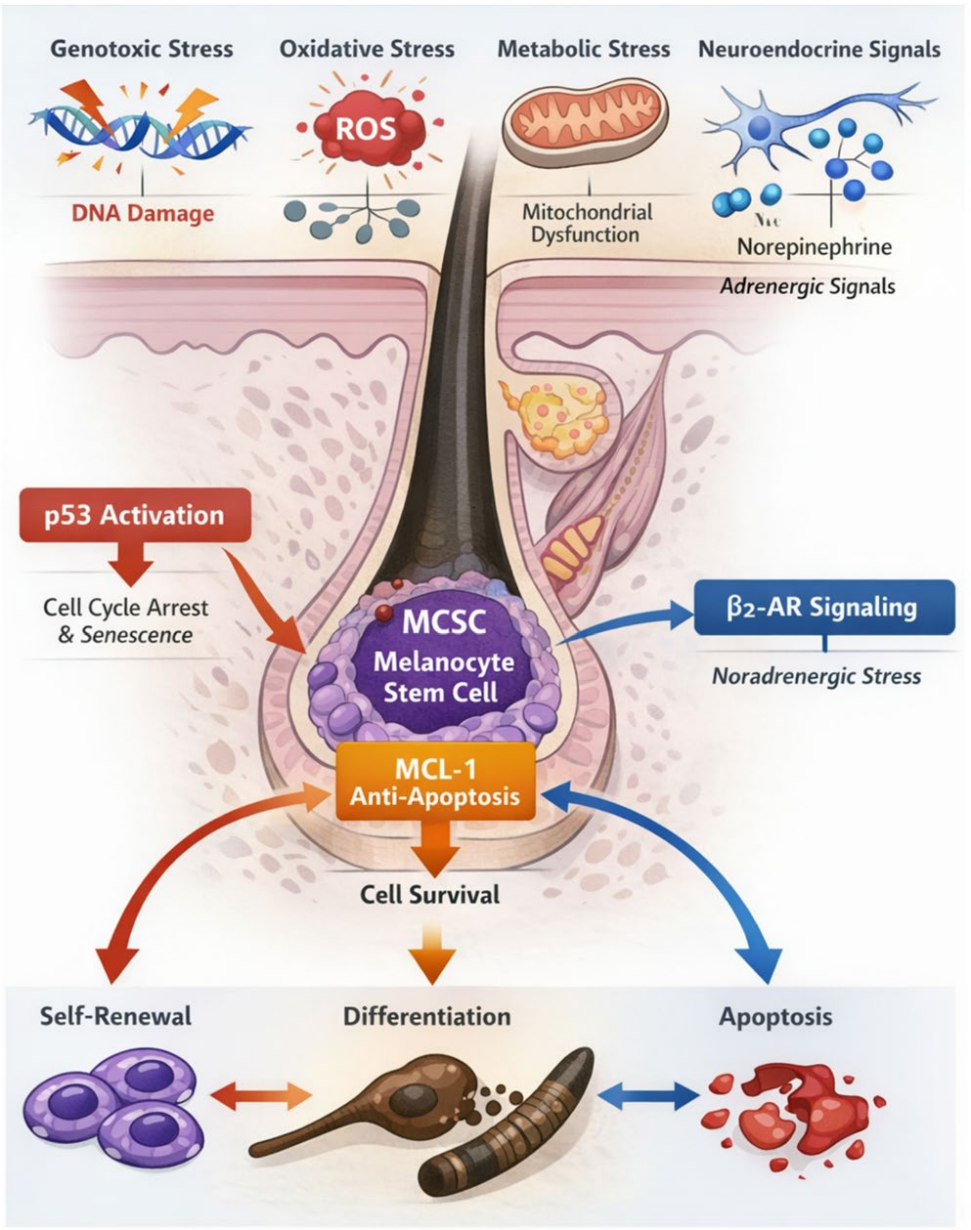
Integration of genotoxic, oxidative, and neuroendocrine signals in melanocyte stem cell regulation. The diagram illustrates how DNA damage (via ATM/ATR), oxidative stress (H_2_O_2_​ accumulation, MSR inactivation), and sympathetic nerve activation (Noradrenaline) converge to force Melanocyte Stem Cells (McSCs) away from self renewal and towards premature differentiation (ectopic pigmentation) or apoptosis. This depletion of the niche leads to irreversible hair graying while serving as a checkpoint against oncogenesis

## The Oxidative and Biochemical Burden

Melanogenesis is inherently a pro-oxidative biosynthetic pathway. The hydroxylation of tyrosine to DOPA and subsequently to melanin generates significant quantities of reactive oxygen species (ROS). In youthful follicles, this endogenous oxidative stress is neutralized by a robust antioxidant network, primarily involving catalase and methionine sulfoxide reductases (MSR A and B). Specifically, MSR enzymes are critical for repairing oxidized methionine residues in key melanogenic proteins, including Tyrosinase and Tyrosinase-related proteins (TRP-1/2).

However, as highlighted in the pathogenesis of premature canities, these defense mechanisms falter with age or extrinsic stress (e.g., smoking, UV radiation). The consequent accumulation of hydrogen peroxide (H_2_O_2_) in the millimolar range leads to the oxidation and inactivation of catalase and MSR enzymes, creating a vicious cycle. This oxidative stress does not merely impair enzymatic function; it inflicts direct DNA damage on the quiescent McSCs residing in the bulge.

## Genotoxic Stress: The “Stemness” vs. Survival Trade Off

How do stem cells respond to this accumulating damage? This is where the paradigm shifts. As elucidated by Inomata et al. (2009) and recently expanded by Mohri et al. (2025), McSCs possess a unique response to genotoxic stress. Unlike other somatic cells that might undergo immediate apoptosis, McSCs activate an ATM/ATR mediated DNA damage response that triggers “ectopic differentiation.” Under genotoxic pressure, McSCs are forced to exit their quiescent state and differentiate into pigment producing melanocytes within the niche, rather than migrating to the bulb. This “stemness” loss is irreversible. Once differentiated, these cells cannot self-renew and are eventually lost. While this leads to stem cell exhaustion and graying, it serves a crucial evolutionary function: it prevents damaged stem cells from persisting, acquiring further mutations, and potentially transforming into melanoma. Thus, hair graying can be interpreted as a tumor-suppressive mechanism a biological “safe mode” that eliminates compromised cells at the cost of pigmentation [1, 2].

## The Neuroendocrine Interface: Stress and the Niche

The popular observation that “stress turns hair white” has now been validated with precise molecular mechanisms, linking the nervous system directly to stem cell fate. The hair follicle bulge is heavily innervated by the sympathetic nervous system. The landmark study by Zhang et al. (2020) demonstrated that acute psychological stress triggers the release of high levels of noradrenaline (norepinephrine). This neurotransmitter binds directly to β_2_-adrenergic receptors on McSCs. Normally, McSC activation is subtle and synchronized with the hair cycle. However, noradrenergic surges bypass these checkpoints, causing massive, hyper-activation of the entire stem cell pool. The McSCs proliferate rapidly, differentiate, and migrate away from the niche, depleting the reservoir. This “fight or flight” response at the cellular level underscores that the sympathetic nervous system is a potent regulator of the stem cell niche, providing a mechanistic link between systemic physiological states and tissue-specific aging [3].

## Survival Signaling and Developmental Signaling

The maintenance of the McSC pool is further governed by a delicate interplay between survival signals (MCL-1) and developmental pathways (Wnt/β-catenin). Chin et al. (2025) recently defined the critical role of the anti-apoptotic protein MCL-1 in this context. MCL-1 acts as a guardian, preventing p53-mediated apoptosis during the proliferative stress of the anagen phase. When MCL-1 levels are compromised, p53 eliminates the stem cells to prevent genomic instability [4]. Concurrently, Wnt signaling is the master regulator coordinating hair growth with pigmentation. For a pigmented hair to grow, Wnt activation must occur simultaneously in both epithelial stem cells (for hair growth) and melanocyte stem cells (for pigment). If Wnt signaling is uncoupled active in the epithelium but silenced in melanocytes the result is the growth of a white hair. This uncoupling is frequently observed in aging and premature graying, suggesting that therapeutic reactivation of Wnt specifically in the melanocyte lineage could theoretically reverse the process [5].

## Clinical Correlates: Autoimmunity and Inflammation

Premature canities frequently overlaps with autoimmune disorders, suggesting an inflammatory dimension. In Alopecia Areata, the phenomenon of Canities Subita (“sudden whitening” or Marie Antoinette syndrome) occurs because the autoimmune attack selectively targets pigmented hair follicles, which express specific melanogenesis-associated autoantigens, while sparing white hairs. This selective preservation of gray hairs implies that the loss of immune privilege in the follicle contributes to pigmentary failure. Similarly, in Vitiligo, the follicular reservoir of melanocytes is often the last to be destroyed but is also the primary source for repigmentation of the epidermis. Depletion of this reservoir in premature graying thus represents a poor prognostic factor for vitiligo repigmentation therapies.

## Therapeutic Frontiers and Future Directions

The reversibility of hair graying remains the “holy grail” of trichology. Current nutritional interventions (Vitamin B12, copper, zinc) are only effective in cases of metabolic deficiency. For true structural restoration of the McSC pool, future therapies must pivot toward niche bioengineering and molecular modulation.

### Wnt Agonists

Pharmacological agents that mimic Wnt/β-catenin signaling could potentially reactivate quiescent McSCs.

### MCL-1 Stabilization

Enhancing MCL-1 expression could protect stem cells from stress-induced apoptosis or differentiation.

### Targeted Delivery

The challenge lies not just in the molecule, but in delivery. Reaching the bulge region requires advanced transdermal carriers or liposomal systems to penetrate the hair follicle infundibulum.

However, a critical caveat remains: the mechanisms driving graying (differentiation upon DNA damage) are evolved defenses against malignancy. Reversing this process forcing damaged stem cells to self-renew instead of differentiating could theoretically increase the risk of follicular tumorigenesis or melanoma. Therefore, the ultimate challenge in treating canities is to decouple pigmentation restoration from oncogenic risk.

In conclusion, premature hair graying is a sentinel of stem cell distress. Whether driven by the accumulation of ROS, neuroendocrine storms, or genotoxic damage, it signifies a breakdown in the homeostatic maintenance of the niche. Deciphering the cross-talk between the immune system, the nervous system, and stem cell checkpoints will likely yield the next generation of therapeutics. These future treatments will aim not just to restore cosmetic color, but to reverse the “seno-differentiation” of aging tissues, offering broader insights into regenerative medicine.

## Data Availability

The data supporting this study’s findings are available from the corresponding author upon reasonable request.
